# Case Report: COVID-19-associated Rhinosinusitis Mucormycosis Caused by *Rhizopus arrhizus*: A Rare but Potentially Fatal Infection Occurring After Treatment with Corticosteroids

**DOI:** 10.4269/ajtmh.21-0359

**Published:** 2021-07-08

**Authors:** Payam Tabarsi, Neda Khalili, Mihan Pourabdollah, Somayeh Sharifynia, Ali Safavi Naeini, Jahangir Ghorbani, Abdolreza Mohamadnia, Zahra Abtahian, Elham Askari

**Affiliations:** 1 Clinical Tuberculosis and Epidemiology Research Center, National Research Institute of Tuberculosis and Lung Diseases (NRITLD), Shahid Beheshti University of Medical Sciences, Tehran, Iran;; 2 School of Medicine, Tehran University of Medical Sciences, Tehran, Iran;; 3 Network of Immunity in Infection, Malignancy and Autoimmunity (NIIMA), Universal Scientific Education and Research Network (USERN), Tehran, Iran;; 4 Chronic Respiratory Diseases Research Center, National Research Institute of Tuberculosis and Lung Diseases (NRITLD), Shahid Beheshti University of Medical Sciences, Tehran, Iran

## Abstract

COVID-19 first emerged in Wuhan, China, in December 2019. Since that time, the frequency of bacterial and fungal coinfections has been continuously increasing. Although invasive pulmonary aspergillosis is being increasingly recognized in association with COVID-19, there is limited information regarding COVID-19-associated mucormycosis. We describe a 50-year-old woman with uncontrolled diabetes who received systemic corticosteroids and remdesevir during her admission for COVID-19. A few days after discharge, the patient was readmitted because of facial swelling and numbness, and a diagnosis of COVID-19-associated rhinosinusitis mucormycosis caused by *Rhizopus arrhizus* (formerly called *Rhizopus oryzae*) was confirmed with sequencing of the internal transcribed spacer region of the ribosomal DNA. This report aimed to address the importance of short-term follow-up for COVID-19 patients who have received systemic corticosteroids, particularly those with predisposing conditions, because early detection and prompt, aggressive treatment are essential for the management of invasive fungal infections.

## INTRODUCTION

Emerging evidence has suggested that patients infected with severe acute respiratory syndrome coronavirus 2 (SARS-CoV-2) may develop bacterial and fungal secondary infections.^1^ Although invasive pulmonary aspergillosis (IPA) has been increasingly recognized in association with COVID-19, especially in critically ill patients hospitalized in the intensive care unit,^2^ there are only a few cases of COVID-19-associated mucormycosis (CAM) available in the literature.^3^ Mucormycosis is a rare, opportunistic, highly fatal fungal infection that typically occurs in individuals with underlying compromising conditions, such as diabetes mellitus, corticosteroid use, hematologic malignancies, neutropenia, solid organ/allogeneic stem cell transplants, primary immunodeficiency, and treatment with immunosuppressants. Nevertheless, such infections can be found in apparently immunocompetent patients, although these cases are extremely rare.^4^ Rhino-orbito-cerebral mucormycosis is considered the most common manifestation of mucormycosis, and it is thought to be acquired via the inhalation of fungal spores into the paranasal sinuses. We describe a patient with uncontrolled diabetes who received dexamethasone and remdesevir for COVID-19 treatment and was readmitted after discharge with a diagnosis of rhinocerebral mucormycosis.

## CASE REPORT

A 50-year-old woman presented with a 3-day history of dry cough, shortness of breath, myalgia, and fatigue. Her medical history included type 2 diabetes mellitus and hypertension, which had been diagnosed 5 years previously. She had also undergone gastric bypass surgery for weight loss 2 years before the current admission. The patient did not use any medications for her diabetes because she assumed that she had controlled blood sugar after her surgery and, possibly, because of her poor compliance with antidiabetic therapy; however, she used two antihypertensive drugs (diltiazem and losartan) on a daily basis. She had no history of tobacco smoking or alcohol consumption. The patient was admitted with a presumptive diagnosis of COVID-19. On admission, she was hemodynamically stable, with blood pressure of 160/100 mmHg and pulse rate of 78 bpm. She had no fever (oral temperature, 37.2°C); however, she had an increased respiratory rate of 32 breaths/min and an oxygen saturation of 88% on room air. Blood tests revealed normal results, and her random plasma glucose level was 224 mg/dL. Reverse-transcription polymerase chain reaction (RT-PCR) indicated SARS-CoV-2, and a diagnosis of COVID-19 pneumonia was confirmed. During hospitalization, remdesivir (200 mg on day 1 and 100 mg on days 2–5) and dexamethasone (6 mg once daily for 10 days) were initiated. After 21 days, the patient was discharged with significant clinical improvement and an oxygen saturation of 95% on room air.

**Figure 1.**
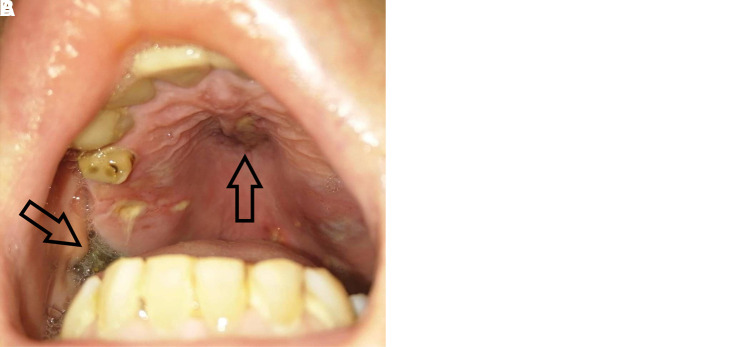
(**A**) Photograph of the patient showing facial swelling, periorbital edema, and erythema (arrows), which are more prominent on the left side. (**B**) Photograph showing necrotic eschars on the palate (arrows). This figure appears in color at www.ajtmh.org.

Five days later, the patient was readmitted because of facial swelling, facial numbness, periorbital edema, and erythema, which were more prominent on the left side of the face, and headache (Figure 1). A careful and thorough physical examination revealed necrotic eschars on the palate and nasal turbinates. Subsequently, the patient underwent nasal endoscopy for further investigation; surgical evaluation was immediately performed for debridement of the necrotic tissues, and multiple biopsies were performed for diagnostic purposes. Laboratory tests performed at admission yielded the following results: random plasma glucose level, 256 mg/dL; hemoglobin A1c, 7.4%; leukocytes, 12.8 × 10^3^/μL (neutrophils, 78%); hemoglobin, 11.4 g/dL; C-reactive protein, 53 mg/l; erythrocyte sedimentation rate, 71 mm/h; and lactate dehydrogenase, 402 U/L. All other laboratory tests were within normal limits (Supplemental Table 1). Additionally, computed tomography of the paranasal sinuses showed severe mucosal thickening of the left maxillary sinus and erosive changes of the maxillary sinus and the left inferior orbital rim, which suggested invasive fungal rhinosinusitis.

**Figure 2.**
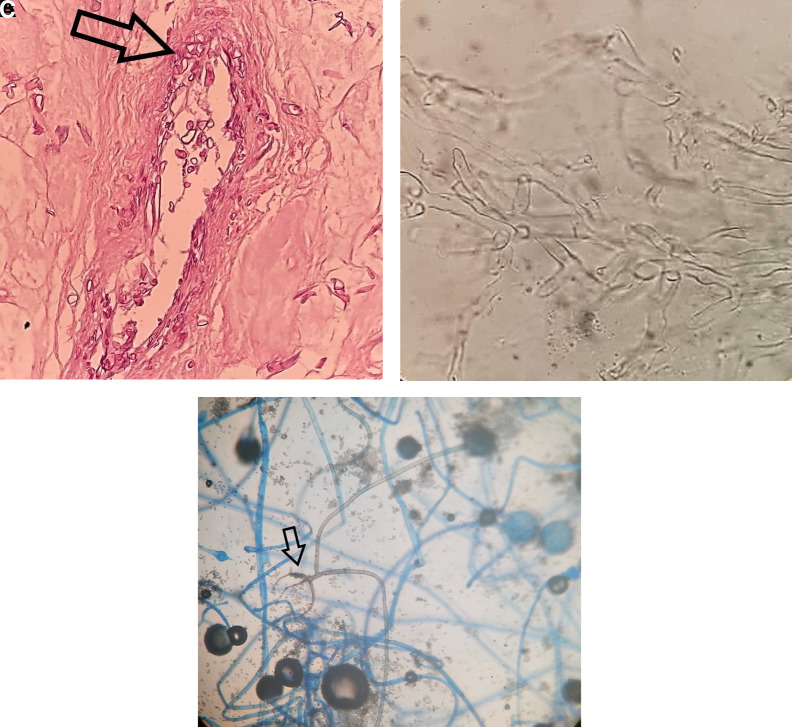
(**A**) Broad, pauciseptate hyphae (arrow) within the wall and lumen of blood vessels (hematoxylin and eosin stain). (**B**) Direct smear with 10% KOH reveals hyaline mycelium and hyphae with distinct characteristics, consistent with Mucorales. (**C**) Lactophenol cotton blue staining of the cultured fungi shows hyphae with nodal rhizoids (arrow) and short sporangiophores with round black sporangia. This figure appears in color at www.ajtmh.org.

Histopathological examination of tissue biopsy samples that were necrotic and suppurative showed broad, pauciseptate hyphae with right-angle branching that were visible within the wall and lumen of blood vessels. A direct smear with 10% KOH revealed hyaline mycelium with hyphae typical of Mucorales. Culture of the tissue on Sabouraud dextrose agar at 25°C and 35°C yielded positive results and showed growth of grayish-white (and later grayish dark brown) colonies with a woolly texture 2 days after incubation. Lactophenol cotton blue staining of the cultured fungi showed hyphae with nodal rhizoids and short sporangiophores with round black sporangia (Figure 2). Finally, sequencing of the internal transcribed spacer (ITS) region of the ribosomal DNA confirmed the diagnosis of infection by *Rhizopus arrhizus* (formerly called *Rhizopus oryzae*).^5^ Sequencing was performed by amplification using ITS1 and ITS4 primers.^6^ Sequences were compared with those of the National Center for Biotechnology Information Genbank database using the BLAST algorithm (https://blast.ncbi.nlm.nih.gov/Blast.cgi) to confirm the identification of the organism as *R. arrhizus*.

The patient received intravenous liposomal amphotericin B during her stay at the hospital. She was finally discharged after 28 days.

## DISCUSSION

At more than 1 year since the emergence of COVID-19 in China, definitive and specific treatment against SARS-CoV-2 is still lacking. Several therapeutic agents, such as corticosteroids and antiviral and immunomodulatory drugs, have been investigated for the management of critically ill patients with COVID-19; however, none has been clinically efficacious.^7^^,^^8^

The administration of systemic corticosteroids has been shown to decrease the mortality of particular subgroups of patients with COVID-19, with the greatest efficacy shown for patients receiving invasive mechanical ventilation.^9^^,^^10^ Nevertheless, treatment with systemic corticosteroids causes immunosuppression, thereby predisposing patients to invasive fungal rhinosinusitis. Corticosteroids may induce hyperglycemia, leading to the activation of immune cells, secretion of proinflammatory cytokines, and development of inflammation.^11^ According to the European Organization for Research and Treatment of Cancer and the Mycoses Study Group Education and Research Consortium (EORTC/MSGERC) consensus, prolonged use of corticosteroids at a therapeutic dose of ≥ 0.3 mg/kg for at least 3 weeks during the past 60 days is considered a risk factor for invasive fungal diseases.^12^ A study by Jeong et al. indicated that corticosteroid use was a predisposing factor for approximately 33% (273/851) of patients with mucormycosis.^13^ Additionally, COVID-19 patients with diabetes are not only at increased risk for severe disease but also more prone to invasive fungal infections.^14^ Diabetes mellitus can alter the body’s immunological response to pathogens by enhancing fungal proliferation and diminishing the phagocytic capacity of host immune cells.^15^ Ketone reductase enzymes in Mucorales, including *Rhizopus* organisms, allow them to thrive in high-glucose, acidic conditions, resulting in the stimulated growth of these organisms in patients with diabetic ketoacidosis.^16^ Our patient presented here, however, did not have diabetic ketoacidosis. Furthermore, IL-6-inhibiting drugs, such as tocilizumab, may cause immune dysregulation and increase the risk of secondary infections without providing substantial clinical benefits for patients with COVID-19.^17^^,^^18^ COVID-19 patients with acute respiratory distress might be susceptible to secondary infections as a result of immune dysregulation.^19^ Patients infected with SARS-CoV-2 have reduced levels of circulating lymphocytes and T-cell subsets, resulting in suboptimal cell-mediated immune responses.^20^ Therefore, it can be anticipated that critically ill patients with COVID-19 are at increased risk for severe invasive fungal infections.

Acute invasive fungal rhinosinusitis is characterized by thrombosis, infarction, and necrosis of involved tissues because of vascular invasion by the fungus, which manifests as black palatal or gingival eschars and/or perforation of the nasal septum. Rhinocerebral mucormycosis usually presents with the acute onset of fever, facial pain, nasal congestion, headache, perinasal swelling, facial numbness, and visual changes, such as diplopia and proptosis. Facial numbness, as seen in the present case, is caused by fifth cranial nerve involvement, which indicates that the infection has spread beyond the sinuses. With the rapid spread of the fungal infection to the brain, obtundation, cranial nerve palsy, cavernous sinus thrombosis, and carotid artery involvement may occur. Cavernous sinus thrombosis is a complication that is usually seen when the fungal infection enters through direct wound contamination and into the oral cavity, thereby involving the mandible. However, palatal ulcers are commonly seen in infections originating from the nose and paranasal sinuses.^15^

The estimated incidence of mucormycosis varies among different continents and countries; in 2019, the reported incidence rates of mucormycosis (per million) were as follows: Europe, from 0.2 cases in Denmark to 95 cases in Portugal; United States, 3.0 cases; Canada, 1.2 cases; Australia, 0.6 cases; and India, 140 cases.^21^ However, the incidence of mucormycosis for patients using dexamethasone without DKA has not yet been reported in the literature.

Based on the available literature, six studies corresponding to 11 patients, including ours, have reported rhino-orbito-cerebral mucormycosis in association with COVID-19. Detailed descriptions of these cases are provided in Table 1. Based on these studies, all patients had diabetes, either previously diagnosed or detected during COVID-19 admission; however, not all patients had received corticosteroids before the initiation of symptoms related to mucormycosis. Importantly, the causative fungus has been identified in only a few studies. Therefore, the most common species causing invasive rhinocerebral mucormycosis could not be determined among these patients. It is worthy to note that although few patients developed symptoms during the hospital stay, others (such as our patient), developed symptoms after being discharged from the hospital for COVID-19 treatment. Therefore, it is important for all physicians to be aware of the fact that invasive fungal infections might occur after patients with COVID-19 have been discharged. Furthermore, patients, particularly those with predisposing conditions, should be informed about the red flag symptoms of invasive mucormycosis.

**Table 1:** Description of previously reported cases of rhino-orbito-cerebral mucormycosis in patients with COVID-19

Study	Patient characteristics	Risk factors	Initiation of symptoms*	Diagnosis	Causative fungus	Outcome
Mehta et al.^24^	60-year-old man with bilateral lid edema	Diabetes + ARDS + methylprednisolone + dexamethasone + tocilizumab	Day 10	Culture	Not identified	Died
Sen et al.^28^	Patient 1: 46-year-old man with ptosis, periocular swelling, and loss of vision	Diabetes	Day 0	Histopathology	Not identified	Alive
	Patient 2: 60-year-old man with ptosis and painful and limited eye movements	Diabetes + methylprednisolone + prednisolone	Day 17†	Culture	Not identified	Alive
	Patient 3: 73-year-old man with ptosis and painful and limited eye movements	Diabetes + prednisolone + dexamethasone	Day 30†	Not proved	Not identified	Alive
	Patient 4: 72-year-old man with periocular swelling, fixed pupil, and loss of vision	Diabetes + prednisolone +	Day 14†	Culture	Not identified	Alive
	Patient 5: 62-year-old man with ptosis, fixed pupil, and loss of vision	Diabetes + dexamethasone	Day 42†	Culture	Not identified	Alive
	Patient 6: 47-year-old man with ptosis, periocular swelling, and loss of vision	Diabetes + dexamethasone	Day 3	Culture	Not identified	Alive (all had vision loss)
Werthman-Ehrenreich et al.^25^	33-year-old woman with altered mental status, ptosis, proptosis, fixed pupil, and ophthalmoplegia	New-onset diabetes + diabetic ketoacidosis	Day 0	Culture	Not identified	Died
Waizel-Haiat et al.^26^	24-year-old woman with left midface pain, left eyelid swelling, proptosis, and maxillary hypoesthesia	New-onset diabetes + diabetic ketoacidosis	Day 0	Culture	*Lichtheimia* spp.	Died
Mekonnen et al.^27^	60-year-old man with right-side proptosis, eyelid swelling, and conjunctival chemosis	Diabetes + systemic corticosteroids	Day 7	NA	*Rhizopus* spp.	Died

* Date of admission for COVID-19 is considered as baseline (Day 0).

† These patients developed symptoms suggestive of mucormycosis after discharge.

Mucormycosis has been diagnosed postmortem for two patients with COVID-19^22^^,^^23^; hence, it is rational to assume that a fatal outcome may be precipitated by invasive fungal infections, such as mucormycosis, in a number of COVID-19 patients with predisposing factors, and that this devastating infection might have been underdiagnosed during the pandemic. Therefore, the early diagnosis of invasive fungal infections, such as rhino-orbito-cerebral mucormycosis, is of critical importance for COVID-19 patients with sinus symptoms, particularly those with underlying diseases and those who have received systemic corticosteroids because prompt, aggressive treatment is essential for an optimal outcome. Early diagnosis and timely management with surgical debridement plus amphotericin B probably contributed to the favorable outcome achieved for the patient presented here. However, four previous case reports of rhino-orbito-cerebral mucormycosis associated with COVID-19 reported that the patients died despite receiving therapy.^24^^–^^27^

In conclusion, defining the characteristics of patients with invasive mucormycosis associated with COVID-19 may help to improve the evaluation of the course of fungal infections in patients with COVID-19 and to determine the most appropriate and applicable preventive measures for highly susceptible COVID-19 patients with the intention of reducing morbidity and mortality. It is important to note that corticosteroids may be associated with potentially fatal side effects for COVID-19 patients, thereby suggesting their potential to be a “double-edged sword.”
